# Comparison of Diagnostic Performance and Image Quality between Topup-Corrected and Standard Readout-Segmented Echo-Planar Diffusion-Weighted Imaging for Cholesteatoma Diagnostics

**DOI:** 10.3390/diagnostics13071242

**Published:** 2023-03-25

**Authors:** Marco Wiesmueller, Wolfgang Wuest, Angelika Mennecke, Matthias Stefan May, Rafael Heiss, Tobit Fuehres, Rolf Janka, Michael Uder, Arnd Doerfler, Frederik Bernd Laun

**Affiliations:** 1 Institute of Radiology, University Hospital Erlangen, Friedrich-Alexander-Universität Erlangen-Nürnberg (FAU), 91054 Erlangen, Germany; 2 Imaging Science Institute, University-Hospital-Erlangen, Friedrich-Alexander-Universität Erlangen-Nürnberg (FAU), 91054 Erlangen, Germany; 3 Institute of Neuroradiology, University-Hospital Erlangen, Friedrich-Alexander-Universität Erlangen-Nürnberg (FAU), 91054 Erlangen, Germany

**Keywords:** diffusion-weighted imaging, DWI, cholesteatoma, turbo spin-echo, TSE, echo-planar imaging, EPI, readout-segmented, topup correction, reduced susceptibility-induced distortions

## Abstract

This study compares the diagnostic performance and image quality of single-shot turbo spin-echo DWI (tseDWI), standard readout-segmented DWI (rsDWI), and a modified rsDWI version (topupDWI) for cholesteatoma diagnostics. Thirty-four patients with newly suspected unilateral cholesteatoma were examined on a 1.5 Tesla MRI scanner. Diagnostic performance was evaluated by calculating and comparing the sensitivity and specificity using histopathological results as the standard of reference. Image quality was independently reviewed by two readers using a 5-point Likert scale evaluating image distortions, susceptibility artifacts, image resolution, lesion conspicuity, and diagnostic confidence. Twenty-five cholesteatomas were histologically confirmed after surgery and originated in the study group. TseDWI showed the highest sensitivity with 96% (95% confidence interval (CI): 88–100%), followed by topupDWI with 92% (95% CI: 81–100%) for both readers. The sensitivity for rsDWI was 76% (95% CI: 59–93%) for reader 1 and 84% (95% CI: 70–98%) for reader 2, respectively. Both tseDWI and topupDWI revealed a specificity of 100% (95% CI: 66–100%) and rsDWI of 89% (95% CI: 52–100%). Both tseDWI and topupDWI showed fewer image distortions and susceptibility artifacts compared to rsDWI. Image resolution was consistently rated best for topupDWI, followed by rsDWI, which both outperformed tseDWI. TopupDWI and tseDWI showed comparable results for lesions’ conspicuity and diagnostic confidence, both outperforming rsDWI. Modified readout-segmented DWI using the topup-correction method is preferable to standard rsDWI and may be regarded as an accurate alternative to single-shot turbo spin-echo DWI in cholesteatoma diagnostics.

## 1. Introduction

Modern radiology plays a major role in the diagnosis of cholesteatoma [1,2], a common non-neoplastic disease in otology, typically found in the middle ear [3]. The reliable depiction and precise anatomic localization of cholesteatoma are essential to prevent severe clinical complications such as the destruction of ossicular structures and adjacent bone followed by the subsequent risk of conductive or sensorineural hearing loss, facial palsy or endocranial complications [4,5,6]. High-resolution computed tomography (CT) is suitable for preoperative diagnosis of osseous disintegration in the middle and inner ear [7,8], but its role for residual or recurrent cholesteatoma foci may be limited [9]. Magnetic resonance imaging (MRI) is appropriate for the pre- and post-surgery assessment using diffusion-weighted imaging (DWI) sequences [10,11,12,13]. Additionally, post-contrast T1-weighted spin-echo sequences may also contribute to the differentiation between cholesteatoma foci and other findings, e.g., granulation or scar tissue [14,15].

In the last decades, several MRI techniques have been proposed for this purpose and evaluated against each other in terms of diagnostic accuracy and image quality. Single-shot turbo spin-echo-based DWI (tseDWI) demonstrated superior performance compared to single-shot echo-planar DWI (EPI-DWI) techniques and therefore is used worldwide in clinical routine [16]. Readout-segmented echo-planar DWI (rsDWI), a successor to conventional EPI-DWI, enables and potentially facilitates image quality and diagnostic accuracy in the temporal region suffering less from geometrical distortions than conventional EPI-DWI [17,18]. Unfortunately, comparisons between rsDWI and tseDWI in cholesteatoma diagnostics led to contradictory results in recent studies. Dudau et al. found a comparable diagnostic performance between both sequences [19], whereas Benson et al. and Wiesmueller et al. found superior diagnostic accuracy and image quality for tseDWI [20,21]. Although in the last-mentioned study, rsDWI showed superior subjective image quality; its diagnostic performance was significantly degraded by higher susceptibility-induced distortions leading to both lower sensitivity and specificity.

Several approaches have been proposed to reduce susceptibility-induced artifacts in EPI-DWI, in particular, the use of higher parallel imaging acceleration factors and dedicated post-processing techniques [22,23]. A recently developed post-processing technique that can reduce susceptibility-induced distortions builds on acquiring pairs of echo-planar images with opposing phase-encoding polarities but otherwise identical settings. From these image pairs, the susceptibility-induced off-resonance field can be estimated as described by Andersson et al. and implemented in Functional MRI of the Brain Software Library (FSL; the “topup” method); and the two images can then be combined into a single corrected one [24,25]. Based on this principle, topup-corrected rsDWI sequences (topupDWI) may show reduced distortion, which may be especially beneficial in the temporal region due to its inhomogeneous magnetic environment.

An improvement in diagnostic performance can thus be anticipated but is not guaranteed, given that the advanced correction algorithm might also introduce artifacts. Hence, this study sought to compare standard uncorrected rsDWI, topupDWI, and tseDWI, focusing on image quality and diagnostic performance in cholesteatoma diagnostics. We hypothesized that topupDWI is able to outperform standard uncorrected rsDWI and may catch up with tseDWI with regard to diagnostic performance in the temporal region.

## 2. Materials and Methods

This prospective study was conducted in accordance with the guidelines of the Declaration of Helsinki and approved by the Institutional Review Board of Friedrich-Alexander Universität Erlangen/Nürnberg.

### 2.1. Study Population and Study Procedure

After the clinical assessment performed by a consultant physician for otorhinolaryngology, patients with a newly suspected unilateral cholesteatoma were included in this prospective study. Patients with contraindications for MRI examination (such as pacemakers, metal fragments, unsuitable implants, or claustrophobia) were excluded. In total, 34 patients were eligible for study participation and underwent MRI to detect suspected cholesteatoma and to evaluate its extension. After MRI examination, all patients underwent surgery, and resultant histopathological findings served as standard of reference for the presence of cholesteatoma foci. All patients signed informed consent prior to MRI examination, and institutional review board approval was obtained.

### 2.2. Image Acquisition

MRI examinations were performed on a 1.5 Tesla (T) MRI scanner (MAGNETOM Aera, Siemens Healthcare GmbH, Erlangen, Germany) using a dedicated 20-channel head and neck coil. The acquisition protocol consisted of routine sequences and two additional rsDWI sequences with opposing phase-encoding directions, as detailed below. 

Our routine cholesteatoma MRI protocol consisted of a T1-weighted sequence in axial-slice orientation with 2 mm slice thickness; a T2-weighted constructive interference steady state (CISS) sequence in axial slice orientation with isotropic 0.8 mm voxel size; and a post-contrast T1-weighted sequence with spectral fat saturation in axial and coronal slice orientation, each acquired with 2 mm slice thickness. Additionally, the entire neurocranium was examined with a T2-weighted fluid attenuation inversion recovery (FLAIR) sequence in axial slice orientation with 5 mm slice thickness. A half-Fourier single-shot turbo spin-echo (HASTE) DWI sequence (tseDWI) of the temporal bone was measured in axial and coronal slice orientation. The tseDWI sequence is the standard sequence used for cholesteatoma diagnostics at our institution and, therefore, part of the routine MRI protocol of the temporal bone.

In addition, a readout-segmented DWI (rsDWI; RESOLVE^®^ = readout of long variable echo trains, Siemens Healthcare GmbH, Erlangen, Germany) sequence of the temporal bone was measured in axial and coronal orientation, initially using right-to-left (RL) phase-encoding direction. Subsequently, rsDWI acquisition was repeated immediately with identical acquisition parameters, except the phase-encoding direction was altered to left-to-right (LR). Both rsDWI data with RL- and LR-phase-encoding direction were utilized for dedicated post-processing algorithms as described in detail below to correct for field inhomogeneity-induced image distortions. A detailed overview of tseDWI and rsDWI sequences are provided in Table 1.

To avoid inhomogeneous image signal intensities by spatially varying coil sensitivity profiles, the vendor-provided prescan normalize option was used for all DWI sequences.

### 2.3. Post-Processing of Readout-Segmented DWI Data

From the two sets of echo-planar images with normal and reversed phase-encoding direction, the susceptibility-induced off-resonance field was estimated with a method similar to that described by Andersson et al. using the tool “topup” of the Functional MRI of the Brain Software Library (FSL) [24,25,26]. The two measurements were combined into a single distortion-corrected image (topupDWI). All images were reviewed, and no image needed to be sorted out due to motion artifacts or other quality issues.

### 2.4. Image Analysis

Image analysis was independently performed by two board-certified radiologists (reader 1 with 7 years of experience and reader 2 with 11 years of experience in head and neck MRI, respectively). Both readers were blinded to additional information on patient status and other images.

First, each reader independently evaluated only tseDWI data in random order. Second, uncorrected rsDWI data with RL phase-encoding direction were assessed a few days later in a different randomized order to avoid any possible recall bias. In a third assessment round, which was again delayed by several days, topupDWI data were evaluated in a different randomized order. Histopathological results were unknown to both readers at the time of each assessment round.

Both readers evaluated the image quality and diagnostic properties for each of the six DWI sequences: tseDWI axial, tseDWI coronal, rsDWI axial, rsDWI coronal, topupDWI axial, topupDWI coronal using the following categories on a five-point Likert scale:

1. Prominence of geometric image distortions in the relevant temporal region (inner ear, middle ear, and outer auditory canal; 1 = very strong, 2 = strong, 3 = medium, 4 = small, and 5 = negligible);
2. Presence of brightly appearing regions that might be mistaken for a true lesion in the temporal region (1 = present and not distinguishable from a true lesion, 2 = present and hardly distinguishable from the true lesion, 3 = present but clearly distinguishable, 4 = hardly present, and 5 = not present);
3. Subjective rating of image resolution (1 = very low, 2 = low, 3 = medium, 4 = good, and 5 = very good);
4. Lesion conspicuity (1 = very bad, 2 = bad, 3 = medium, 4 = good, 5 = very good).

Regarding subjective diagnostic confidence, the readers rated the two tseDWI (axial and coronal combined) datasets, the two rsDWI (axial and coronal combined) datasets, and the two topupDWI (axial and coronal combined) datasets in a fourth assessment round:

5. Diagnostic confidence (1 = very low, 2 = low, 3 = medium, 4 = high, and 5 = very high);
6. For every single dataset (tseDWI, rsDWI, and topupDWI), reader 1 and reader 2, respectively, had to decide whether a cholesteatoma foci was present or not (yes/no). Decision criteria were the signal intensity of adjacent brain tissue compared to an assumed cholesteatoma foci. The decision was “yes” if a temporal lesion revealed a hyperintense signal intensity.

In a fifth assessment cycle, each reader rated simultaneously the following DWI dataset combinations: tseDWI versus rsDWI; tseDWI versus topupDWI; rsDWI versus topupDWI. Each rated DWI dataset consisted of both axial and coronal reconstruction. The assessment was performed regarding

7. Lesion conspicuity when comparing tseDWI versus rsDWI (2 = much better with tseDWI, 1 = better with tseDWI, 0 = equal, −1 = better with rsDWI, and −2 = much better with rsDWI);
8. Lesion conspicuity when comparing tseDWI versus topupDWI (2 = much better with tseDWI, 1 = better with tseDWI, 0 = equal, −1 = better with topupDWI, and −2 = much better with topupDWI);
9. Lesion conspicuity when comparing topupDWI versus rsDWI (2 = much better with topupDWI, 1 = better with topupDWI, 0 = equal, −1 = better with rsDWI, and −2 = much better with rsDWI);
10. Subjective diagnostic confidence when comparing tseDWI versus rsDWI (2 = much better with tseDWI, 1 = better with tseDWI, 0 = equal, −1 = better with rsDWI, and −2 = much better with rsDWI);
11. Subjective diagnostic confidence when comparing tseDWI versus topupDWI (2 = much better with tseDWI, 1 = better with tseDWI, 0 = equal, −1 = better with topupDWI, and −2 = much better with topupDWI);
12. Subjective diagnostic confidence when comparing topupDWI versus rsDWI (2 = much better with topupDWI, 1 = better with topupDWI, 0 = equal, −1 = better with rsDWI, and −2 = much better with rsDWI).

### 2.5. Statistical Analysis

Statistical analysis was performed independently for each reader and each image dataset, calculating the sensitivity and specificity with 95% confidence intervals using the Clopper–Pearson method separately for the two groups of patients with histologically proven cholesteatomas and no cholesteatomas. Additionally, the positive predictive value (PPV) and negative predictive value (NPV) were computed. Furthermore, McNemar’s test was performed to compare the three DWI sequences regarding diagnostic accuracy. Inter-rater agreement was assessed by calculating Cohen’s kappa value (κ); κ was interpreted as follows: 0 < κ ≤ 0.2 indicated slight agreement, 0.2 < κ ≤ 0.4 indicated fair agreement, 0.4 < κ ≤ 0.6 indicated moderate agreement, 0.6 < κ ≤ 0.8 indicated substantial agreement, 0.8 < κ ≤ 1.0 indicated almost perfect agreement, and κ = 1 indicated perfect agreement. A comparison between tseDWI, rsDWI, and topupDWI dataset ratings was performed using non-parametric Friedman test with pairwise Dunn–Bonferroni post-hoc tests. Significance was accepted for all *p*-values of less than 0.05. Statistical analysis was performed using the SPSS Statistics software version 24 (IBM Corporation, Armonk, NY, USA).

## 3. Results

### 3.1. Patient Population

The study population consisted of 17 female and 17 male patients, respectively. The mean age was 53 ± 18 years (18–85 years). Twenty-five of the 34 included patients (73.5%) were finally diagnosed with unilateral cholesteatoma after surgical excision and following histopathological confirmation. In 9 patients (26.5%), no histopathological evidence of cholesteatoma could be found. The mean lesion diameter was 0.7 ± 0.2 cm (median: 0.6 cm, range: 0.3–1.8 cm).

### 3.2. Diagnostic Performance

Regarding the 25 cholesteatoma patients, reader 1 found 24 cholesteatomas with tseDWI, 20 with rsDWI, and 23 with topupDWI. In total, 19/25 (76%) were concordantly found with all three DWI sequences, 23/25 (92%) were consistently found with topupDWI and tseDWI, 19/25 (76%) with rsDWI and tseDWI, and 19/25 (76%) rsDWI and topupDWI. The sensitivity for tseDWI was 96% [95% confidence interval (CI): 88–100%], for rsDWI 76% (95% CI: 59–93%), and for topupDWI 92% (95% CI: 81–100%). 

Reader 2 correctly found 24 cholesteatomas with tseDWI, 21 with rsDWI and 23 with topupDWI. Furthermore, 20/25 (80%) were concordantly found with all three DWI sequences. In addition, 23/25 (92%) were consistently found with tseDWI and topupDWI, 21/25 (84%) with rsDWI and tseDWI, and 20/25 (80%) with rsDWI and topupDWI. The sensitivity for tseDWI was 96% (95% CI: 88–100%), for rsDWI 84% (95% CI: 70–98%), and for topupDWI 92% (95% CI: 81–100%). For both readers, no significant differences were found between tseDWI and topupDWI (McNemar’s *p* = 1 for both readers) and between topupDWI and rsDWI (reader 1: McNemar’s *p* = 0.13; reader 2: McNemar’s *p* = 0.62). Comparison between tseDWI and rsDWI revealed a significant difference for reader 1 (McNemar’s *p* = 0.07), whereas no significant difference was observed for reader 2 (McNemar’s *p* = 0.25). 

Regarding the 9 patients without cholesteatoma, reader 1 negatively diagnosed all 9 patients with tseDWI and topupDWI, leading to specificities of 100% (95% CI: 66–100%). In addition, 8/9 patients were correctly classified as negative by rsDWI, yielding a specificity of 89% (95% CI: 52–100%). On the other hand, reader 2 correctly classified all 9 patients without cholesteatoma with rsDWI, topupDWI, and tseDWI, leading to specificities of 100% for all three modalities (95% CI: 66–100%). Therefore, due to the high number of correctly diagnosed patients without cholesteatoma in all three modalities for both readers, McNemar’s test is not reasonably applicable in this situation. 

The PPV for tseDWI and topupDWI was 1 for readers 1 and 2, respectively. PPV for rsDWI was 0.95 for both readers. The negative predictive value (NPV) for tseDWI was 0.90 (both readers). NPV for topupDWI was 0.82 (both readers), and rsDWI showed a lower NPV (reader 1: 0.62; reader 2: 0.69). The overall agreement between both readers concerning the presence of a cholesteatoma was 100% (κ = 1) for tseDWI and topupDWI, respectively. Moreover, 91% (κ = 0.82) overall agreement was found for rsDWI.

### 3.3. Likert Score Ratings

**Figure 1:**
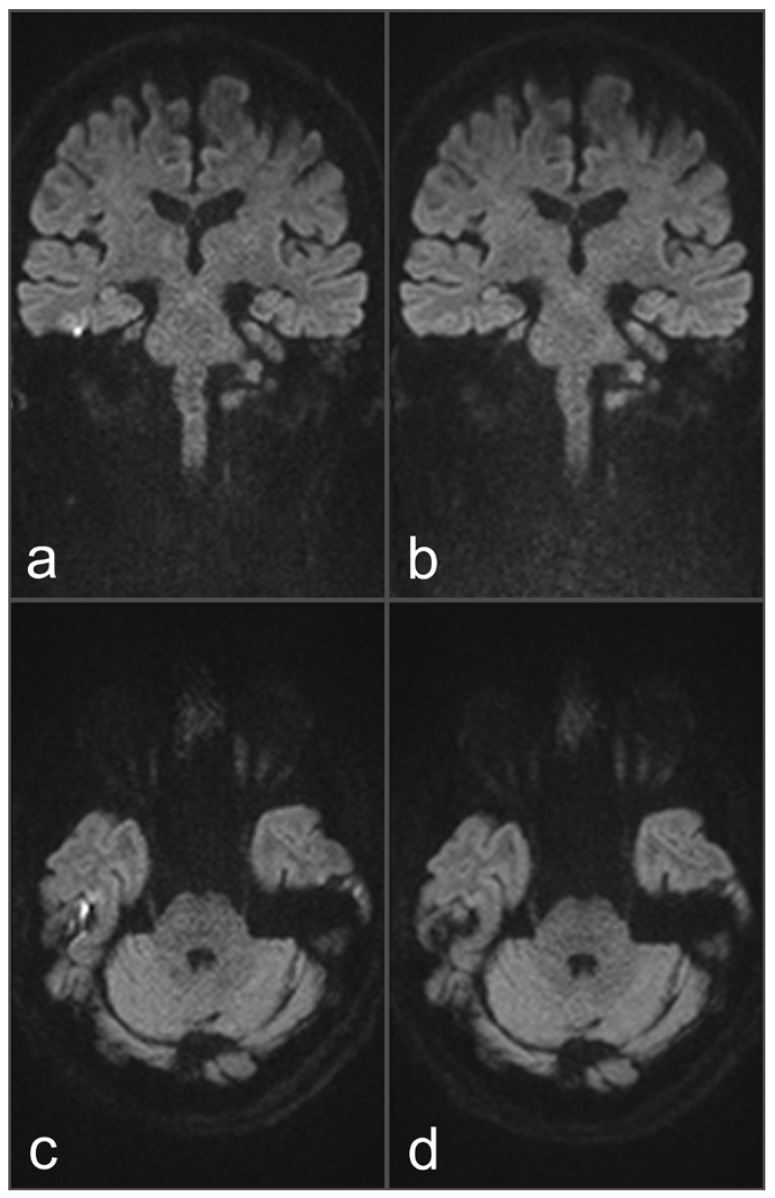
Images of a 34-year-old patient with a suspected cholesteatoma on the right side. (**a**) and (**c**) uncorrected rsDWI (coronal and axial slice orientation). (**b**) and (**d**) topup-corrected rsDWI (coronal and axial slice orientation). Field inhomogeneities at the height of the upper temporal bone generate a brightly appearing lesion in the uncorrected rsDWI images which might be mistaken for a cholesteatoma lesion. Notably, the lesion shows a geometrical distortion on axial slice orientation, whereas a rounded appearance could be identified on coronal image. After topup-correction, the brightly appearing lesion on the right side disappeared completely, unmasking the lesion as an artifact. The “no cholesteatoma” diagnosis was confirmed after surgery with negative histopathological proof.

**Figure 2:**
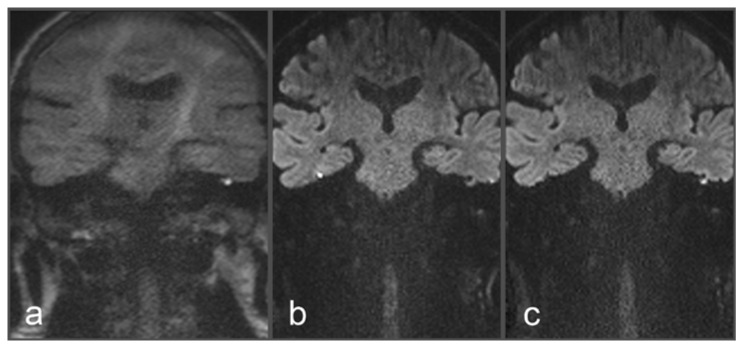
Images of a 48-year-old patient with a suspected cholesteatoma on the left side. (**a**) tseDWI. (**b**) uncorrected rsDWI. (**c**) topup-corrected rsDWI (each sample image in coronal slice orientation). Both tseDWI and topup-corrected rsDWI show a small hyperintense lesion in the upper middle ear on the left side, which could be confirmed as a cholesteatoma after surgery and histopathological examination. In contrast, uncorrected rsDWI was not able to delineate the cholesteatoma on the left side with comparable hyperintense signal intensity and shows a strong geometrical distortion of the lesion. Additionally, uncorrected rsDWI demonstrates two additional lesions: punctual on the left lateral side and brightly appearing on the right side; both were valued as artifacts due to their absence in tseDWI and topup-corrected rsDWI, respectively.

**Figure 3:**
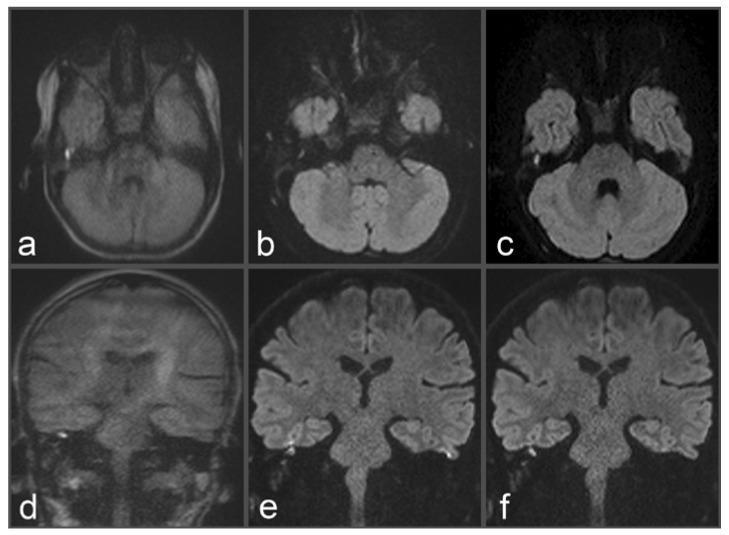
Images of a 54-year-old patient with a suspected cholesteatoma on the right side. (**a**) and (**d**) tseDWI (axial and coronal slice orientation). (**b**) and (**e**) uncorrected rsDWI (axial and coronal slice orientation). (**c**) and (**f**) top-up-corrected rsDWI (axial and coronal slice orientation). tseDWI images show an elongated hyperintense middle-ear lesion on the right side, which could be confirmed as a cholesteatoma after histopathological examination. The lesion is hardly visible in both uncorrected rsDWI images (especially on the axial images) and exhibits a geometrical distortion with a bent-like appearance on both slice orientations. Additionally, two punctiform hyperintensities are visible on both sides at the height of the temporal cortex. Both hyperintensities are missing on the tseDWI and the topup-corrected rsDWI images, strongly indicating that these hyperintensities represent artifacts. On the topup-corrected rsDWI images, the cholesteatoma on the right side exhibits a better contrast compared to uncorrected rsDWI, and geometrical distortions are comparably minor. Additionally, topup-corrected rsDWI does not show the above-described likely false-positive lesions at the border zone between bone and brain tissue.

Observed frequencies and mean differences of Likert scores per category and per reader are summarized in Table 2a,b. For tseDWI, both readers observed minor or negligible geometric distortions, and the presence of bright-appearing regions that might be mistaken for a true lesion was hardly or not present, respectively. TopupDWI was rated second best regarding the above-named evaluation categories, whereas rsDWI was outperformed by both tseDWI and topupDWI. Furthermore, rsDWI outperformed tseDWI only in terms of the subjective image resolution; notably, topupDWI was even rated slightly better than rsDWI by both readers. Finally, both tseDWI and topupDWI outperformed rsDWI in terms of lesions’ conspicuity and diagnostic confidence. Representative images are provided in Figure 1, Figure 2 and Figure 3. 

Regarding evaluation categories 7–12, tseDWI and topupDWI always performed equally or even better than rsDWI. Comparison between topupDWI and tseDWI for evaluation categories 7–12 did not reveal a preference; tseDWI was preferred over topupDWI in less than 50% by both readers. Table 3 summarizes the scoring results from categories 7–12.

A substantial to almost perfect inter-rater agreement was observed for all evaluation categories. Kappa-values for categories 1–5 are summarized in Table 3 and 7–12 in Table 4, respectively. Additionally, we performed a reader-specific comparison between the scoring results of evaluation categories 1–5 derived from tseDWI, rsDWI, and topupDWI with pairwise post-hoc tests. Notably, for both readers, each category revealed a highly significant difference (*p* < 0.001) between the three MRI modalities using the Friedman test in axial and coronal slice orientation. A detailed presentation of all post-hoc test results is absent due to the high number of test situations. Instead, we refer to Table 5 for a detailed overview of all post-hoc test results.

## 4. Discussion

In our study, topup-corrected rsDWI delivered a superior diagnostic performance with respect to the detection of cholesteatoma foci, geometric image distortions, the presence of false positive findings, subjective image resolution, lesions conspicuity, and diagnostic confidence in comparison to uncorrected rsDWI. In addition, tseDWI served as a radiological reference and showed superior sensitivity and specificity, although topupDWI and tseDWI performed almost equally with few benefits for tseDWI. So far, and to our best knowledge, no other study has evaluated the impact of topup-corrected rsDWI in cholesteatoma diagnostics.

TopupDWI, a method to further decrease the image distortions in rsDWI, indeed provided less geometric image distortion and simultaneously exhibited less brightly appearing regions, which might be mistaken for a cholesteatoma.

The topup correction method has been widely applied in diffusion MRI and is, for example, part of the minimal preprocessing pipeline for the Human Connectome Project [27]. As the distortions in non-segmented and segmented EPI are similar in nature, the correction method can be applied in both cases. Naturally, distortion correction is of high importance in diffusion tensor imaging tractography and connectomics studies [28,29,30]. Moreover, it is also commonly applied successfully in a wide range of studies that make use of advanced diffusion techniques such as tract-based spatial statistics (TBSS), q-space trajectory imaging (qti), or diffusion kurtosis imaging [31,32,33]. At high resolutions and high field strengths, it may become of special importance [34,35]. The geometric distortions themselves obviously are very important to consider in all these cases. This is also true in our work, as the correction of the distortions allows one to better delineate the actual position of the lesions. However, one special aspect of our work is that the correction of the intensity that goes along with the distortion is highly relevant because it avoids the appearance of false positive lesions.

As stated in the introduction, recent studies dealing with rsDWI for cholesteatoma diagnostics are ambiguous with respect to its diagnostic performance; hence it remained doubtful if rsDWI could be regarded as an eligible alternative DWI sequence for clinical routine compared to non-EPI DWI. In our opinion, uncorrected rsDWI should not be considered equal to tseDWI in terms of diagnostic accuracy and confidence. This statement is confirmed by our results in this study, which are in accordance with a study by Benson et al. [20]. In contrast, topupDWI showed an enhanced diagnostic performance and almost caught up with tseDWI. Compared to tseDWI, topupDWI missed out only one cholesteatoma foci by both readers, whereas rsDWI missed out four cholesteatomas in the assessment of reader 1 and three cholesteatomas by reader 2, respectively. In addition, with tseDWI, we observed one false-negative finding in our collective. This discrepancy likely originated in a very small cholesteatoma foci, confirmed by histopathological results. In the literature, cholesteatoma foci <2 mm are considered to be frequently missed out with tseDWI [36]. The additional missed-out cholesteatomas for topupDWI and rsDWI may be explained by the longer TE time value used for tseDWI, which might increase the detection rate due to an increased T2-weighting. 

In contrast, the enhanced depiction rate by topupDWI indicates that the better diagnostic performance of tseDWI is not only caused by the potentially more optimal longer TE values of the tseDWI protocol. In fact, we believe the better performance of topupDWI compared to rsDWI is based on the enhanced image quality with fewer distortions and fewer false-positive hyperintensities. Furthermore, the higher sensitivity and specificity for topupDWI concordantly interact with the promising subjective ratings of both readers, who certified superior ratings compared to rsDWI. Notably, topupDWI combines an encouraging diagnostic performance with a desirable high spatial resolution for the precise anatomical depiction of cholesteatoma foci in the temporal bone. Furthermore, tseDWI lacks a clear visualization of anatomical landmarks, which can be regarded as one major drawback of this otherwise very robust DWI sequence. Especially in patients with residual cholesteatomas, a reliable depiction of anatomical landmarks would potentially help the surgeon for second-look surgery.

We conclude that topupDWI showed very promising results in terms of diagnostic accuracy and subjective image quality for cholesteatoma diagnostics. Thus, topupDWI is recommended instead of uncorrected rsDWI for cholesteatoma diagnostics and may be regarded as a potential alternative DWI sequence to single-shot tseDWI.

Some limitations of our study must be acknowledged. We did not perform quantitative evaluations of the signal-to-noise ratio (SNR) nor other quantitative measures of image quality, such as the contrast-to-noise ratio (CNR). Ideally, given that we used multi-channel coils, an evaluation of the SNR would have featured a repetitive acquisition of each dataset [37], which would have prolonged the total scan time. Moreover, we did not consider computed b-value images [38] and did not perform a dedicated quality assurance program [39]. Furthermore, we investigated only 25 patients with proven unilateral cholesteatoma. For the general validity of our conclusions, a larger number is needed in future studies.

## 5. Conclusions

The use of topupDWI is recommended in cholesteatoma diagnostics and can be regarded as a potential alternative DWI sequence to single-shot tseDWI. However, an uncorrected rsDWI sequence is not recommended due to lower diagnostic accuracy.

## Figures and Tables

**Table 1:** DWI sequence parameters.

Sequence	tseDWI	rsDWI (RL)	rsDWI (LR)
Repetition time [ms]	2000	4000	4000
Echo time [ms]	103	65 (and 90 for phase correction scan)	65 (and 90 for phase correction scan)
Voxel size [mm³]	1.1 mm³ × 1.5 mm³ × 3 mm³	1.4 mm³ × 1.4 mm³ × 3 mm³	1.4 mm³ × 1.4 mm³ × 3 mm³
Field of view [mm]	220	230	230
Field of view in phase direction	100%	65%	65%
Phase direction	Anteroposterior (axial), right to left (coronal)	Right-to-left (axial and coronal)	Left-to-right (axial and coronal)
Phase resolution	75%	100%	100%
Partial Fourier	50% (phase)	87.5% (readout)	87.5% (readout)
Matrix	192 × 144	160 × 104	160 × 104
Slice distance	10%	10%	10%
No. of slices	13 (axial) 11 (coronal)	13 (axial) 11 (coronal)	13 (axial) 11 (coronal)
Parallel imaging	GRAPPA × 2	GRAPPA × 2	GRAPPA × 2
Bandwidth, Hz/pixel	554	977	977
Echo spacing, ms	4.48	0.36	0.36
Readout segments	1	5	5
Phase Partial Fourier	0.5	1	1
Read Partial Fourier	1	7/8	7/8
Flip angle	150°	180°	180°
b-values [s/mm^2^]	1000	0, 1000	0, 1000
Averages	10	1 (for b = 0 s/mm²), 2 (for b = 1000 s/mm²)	1 (for b = 0 s/mm²), 1 (for b = 1000 s/mm²)
Diffusion mode	3D diagonal	4-scan trace	4-scan trace
Diffusion scheme	bipolar	bipolar	bipolar
Acquisition time [min]	4:22 (axial), 3:42 (coronal)	3:06	1:50

**Table 2:** **a.** Likert score evaluation (categories 1–5) per reader. **b.** Mean differences between readers 1 and 2 of Likert score evaluation (categories 1–5).

a							
		tseDWI (Likert Scores 4 and 5)		rsDWI (Likert Scores 4 and 5)		topupDWI (Likert Scores 4 and 5)	
Category	Coronal/Axial	Reader 1	Reader 2	Reader 1	Reader 2	Reader 1	Reader 2
Geometric image distortion	Coronal	100%	100%	0%	0%	85%	88%
Geometric image distortion	Axial	100%	100%	0%	0%	85%	88%
Bright-appearing region	Coronal	100%	100%	0%	0%	85%	85%
Bright-appearing region	Axial	100%	100%	0%	0%	88%	88%
Subjective image resolution	Coronal	0%	0%	88%	94%	94%	97%
Subjective image resolution	Axial	0%	0%	74%	76%	85%	85%
Lesion conspicuity	Coronal	91%	97%	6%	0%	82%	88%
Lesion conspicuity	Axial	97%	100%	29%	21%	85%	88%
Diagnostic confidence	Coronal/Axial combined	97%	100%	15%	15%	56%	65%
**b**							
		**tseDWI and rsDWI** **(Positive** **→ tseDWI Better)**		**tseDWI and topupDWI** **(Positive** **→ tseDWI Better)**		**topupDWI and rsDWI** **(Positive** **→ topupDWI Better)**	
**Category**	**Coronal/Axial**	**Reader 1**	**Reader 2**	**Reader 1**	**Reader 2**	**Reader 1**	**Reader 2**
Geometric image distortion	Coronal	2.9	2.9	0.7	0.7	2.2	2.1
Geometric image distortion	Axial	2.6	2.6	0.6	0.6	2	2
Bright-appearing region	Coronal	2.5	2.5	0.6	0.6	1.9	1.9
Bright-appearing region	Axial	2.6	2.6	0.5	0.5	2.1	2.1
Subjective image resolution	Coronal	−2.1	−2.3	−2.3	−2.3	0.2	0
Subjective image resolution	Axial	−1.9	−2	−2.1	−2.1	0.2	0.1
Lesion conspicuity	Coronal	1.9	2.2	0.4	0.5	1.6	1.7
Lesion conspicuity	Axial	1.4	1.6	0.6	0.6	0.8	0.9
Diagnostic confidence	Coronal/Axial combined	1.6	1.7	0.9	0.9	0.7	0.8

**Table 3:** Cohen’s kappa values (κ) represent the inter-rater agreement (evaluation categories 1–5).

Category	Coronal/Axial	tseDWI	rsDWI	topupDWI
Geometric image distortion	Coronal	0.77	0.76	0.74
Geometric image distortion	Axial	0.85	0.79	0.84
Bright-appearing region	Coronal	0.87	0.6	0.81
Bright-appearing region	Axial	0.84	0.64	0.80
Subjective image resolution	Coronal	0.74	0.72	0.71
Subjective image resolution	Axial	0.84	0.80	0.78
Lesion conspicuity	Coronal	0.68	0.64	0.77
Lesion conspicuity	Axial	0.75	0.77	0.80
Diagnostic confidence	Coronal/Axial combined	0.66	0.78	0.73

**Table 4:** Likert scoring results for evaluation of categories 7–12 per reader and inter-rater agreement (κ values). Each listed percentage value refers to a better or much better rating of the first named DWI sequence in brackets (e.g., tseDWI vs. rsDWI).

Category	Better or Much Better with First Named Sequence—Reader 1	Better or Much Better with First Named Sequence—Reader 2	κ
Lesion conspicuity (tseDWI vs. rsDWI)	94%	100%	0.61
Lesion conspicuity (tseDWI vs. topupDWI)	44%	35%	0.82
Lesion conspicuity (topupDWI vs. rsDWI)	85%	88%	0.72
Subjective diagnostic confidence (tseDWI vs. rsDWI)	97%	100%	0.71
Subjective diagnostic confidence (tseDWI vs. topupDWI)	38%	50%	0.65
Subjective diagnostic confidence (topupDWI vs. rsDWI)	71%	79%	0.68

**Table 5:** Reader-specific comparison between scoring results of evaluation categories 1–5 derived from tseDWI, rsDWI, and topupDWI with pairwise post-hoc tests.

Reader 1				
Likert Categories	*p* (Comparison between tseDWI, topupDWI and rsDWI)	*p* (Pairwise Comparison between tseDWI and topupDWI)	*p* (Pairwise Comparison between tseDWI and rsDWI)	*p* (Pairwise Comparison between rsDWI and topupDWI)
Geometric image distortion (axial orientation)	<0.001	0.1	<0.001	<0.001
Geometric image distortion (coronal orientation)	<0.001	0.06	<0.001	<0.001
Bright-appearing region (axial orientation)	<0.001	0.27	<0.001	<0.001
Bright-appearing region (coronal orientation)	<0.001	0.1	<0.001	<0.001
Subjective image resolution (axial orientation)	<0.001	<0.001	<0.001	1
Subjective image resolution (coronal orientation)	<0.001	<0.001	<0.001	1
Lesion conspicuity (axial orientation)	<0.001	0.03	<0.001	0.003
Lesion conspicuity (coronal orientation)	<0.001	0.44	<0.001	<0.001
Diagnostic confidence (axial and coronal combined)	<0.001	0.006	<0.001	0.002
**Reader 2**				
Geometric image distortion (axial orientation)	<0.001	0.075	<0.001	<0.001
Geometric image distortion (coronal orientation)	<0.001	0.033	<0.001	<0.001
Bright-appearing region (axial orientation)	<0.001	0.24	<0.001	<0.001
Bright-appearing region (coronal orientation)	<0.001	0.16	<0.001	<0.001
Subjective image resolution (axial orientation)	<0.001	<0.001	<0.001	1
Subjective image resolution (coronal orientation)	<0.001	<0.001	<0.001	1
Lesion conspicuity (axial orientation)	<0.001	0.03	<0.001	<0.001
Lesion conspicuity (coronal orientation)	<0.001	0.24	<0.001	<0.001
Diagnostic confidence (axial and coronal combined)	<0.001	0.004	<0.001	0.002

## Data Availability

All data generated and analyzed during this study are included in this published article. Raw data supporting the findings of this study are available from the corresponding author on request.
